# Supplementary material for: HIPI: Spatially resolved multiplexed protein expression inferred from H&E WSIs
Source: PLoS Comput Biol. 2024 Sep 30;20(9):e1012501. doi: 10.1371/journal.pcbi.1012501 (PMC11476684; doi:10.1371/journal.pcbi.1012501)
Supplement: S1 Appendix — More details on the method and implementation, together with additional figures. (PDF) [file pcbi.1012501.s001.pdf]

HIPI: Spatially Resolved Multiplexed Protein Expression Inferred from H&E WSIs

Ron Zeira[1], Leon Anavy[1], Zohar Yakhini[1], Ehud Rivlin[1], and Daniel Freedman[1]

[1]Verily AI
{*ronzeira,lanavy,zohary,ehud,danielfreedman*}*@verily.com*

September 13, 2024

S1 Supplementary Methods

S1.1 Preprocessing CyCIF Samples

S1.1.1 Aligning H&E and CyCIF

The same tissue section cannot be used to produce both histological stained image and immunofluorescence image. Therefore, usually multiple thin serial sections of the same tissue are generated and subjected to staining or immunofluorescence imaging. However, adjacent slices and their corresponding images are not perfect replicates of one another. First, there are some physical differences between sections caused by natural tissue structure as well as deformations caused by the manual resection. Second, positioning of the slices for scanning and artifacts created by the imaging technology result in unaligned images of adjacent sections.

We employ a heuristic alogirthm to align H&E and CyCIF images taken from consecutive tissue sections. Since the placement and orientation of the tissue varies between sections, we first find a global registration of the two images. To accomplish this, we apply a linear affine alignment on down-sampled grayscale versions of the H&E and CyCIF images. Because serial sections of the tissue are not exact copies of one another, we then refine our registration on small local tiles. We align overlapping local tiles using a non-linear registration technique to account for small tissue differences.

Let $I \in \mathbb{R}^{n_I \times m_I \times 3}$ and $C \in \mathbb{R}^{n_C \times m_C \times d_C}$ be the H&E and CyCIF images respectfully. In addition, we assume the CyCIF data was processed to produce cell-level expression values. Let $W \in \mathbb{N}^{l \times 2}$ be the coordinates of l cells in the CyCIF image (i.e. for each cell i, $W[i] \in [0 \ldots n_C] \times [0 \ldots m_C]$) and let $Z \in \mathbb{R}^{l \times d_C}$ be the expression values for each cell. Our goal is to find translated coordinates $W' \in \mathbb{N}^{l \times 2}$ of the cell, where $W'[i] \in [0 \ldots n_I] \times [0 \ldots m_I]$ is now the approximated position of the cell in the H&E image.

We apply the following alignment heuristic (Figure 1b): First, we grayscale the H&E image. Then, we resize the CyCIF image to around the H&E scale and use one of the auto autofluorescence channels to grayscale the CyCIF image. We find a global affine alignment transformation on downsampled versions of the two images and translate the cell coordinates accordingly. Finally, we perform local non-linear alignment of images tiles using both affine and B-spline registration. We transform the coordinates of the cells to the H&E image by finding an approximate inverse transformation.

S1.1.2 Extracting tile Level Expression

Let $W' \in \mathbb{N}^{l \times 2}$ be the coordinates of l cells in the H&E image and let $Z \in \mathbb{R}^{l \times d_C}$ be the expression values for each cell. We log transform the expression values and normalize each protein by its mean and standard deviation to obtain Z' (Figure 1c). We extract strided image tiles of size w and the corresponding

cells in each tile. For each such tile with a predefined minimum number of cells, we calculate the mean expression for each protein of cells within a tile (Figure 1c). We denote by $\mathcal{P} = (I_i, z_i)$, the set of H&E image tiles and corresponding expression vectors pairs.

S1.2 Model for Predicting Expression from H&E tiles

Let $\mathcal{F} : [0,1]^{w \times w \times 3} \to \mathbb{R}^d$ be a function that transforms a $w \times w$ H&E image to a d-dimensional feature vector and let $\mathcal{R} : \mathbb{R}^d \to \mathbb{R}^{d_C}$ be a function that maps a d-dimensional feature vector to a normalized tile expression of d_C proteins. Our goal is to train a model $\mathcal{M} = \mathcal{R} \circ \mathcal{F} : [0,1]^{w \times w \times 3} \to \mathbb{R}^{d_C}$ that predicts a normalized tile expression of d_C proteins from a $w \times w$ H&E image and minimizes the squared error $\mathbb{E}_{I,z \in \mathcal{P}}(\|\mathcal{M}(I) - z\|)$.

Self-Supervised learning (SSL) methods, which leverage large amounts of unlabeled data to pre-train meaningful feature extractors, have been shown to out-performs supervised pre-training on multiple tasks [1]. This approach is especially compelling in the field of digital pathology where there are large data sets of unlabeled slides in comparison to limited number of annotated data sets. Indeed, SSL methods for digital pathology have been shown as meaningful feature extractors for multiple downstream tasks even in the face of limited amount of labeled data [2, 3].

Here we use an SSL model pre-trained on publicly available pathology images and use it as a fixed feature extractor $\mathcal{F}$ [3]. Specifically, we use a Vision Transformer (ViT) trained using DINO which gave good results on various task in a recent benchmark [4, 1]. On top of the feature extractor, we train a regressor $\mathcal{R}$ modeled as a fully connected multi-layer perceptron. We call our combined model HIPI.

For reference, we also train two baseline models. The first baseline model extracts the mean color intensities and uses a single linear layer to predict the expression. Formally, $\mathcal{F}(I)_k = \frac{1}{w^2} \sum_{ij} I_{ijk}$ and $\mathcal{R}(v) = Av$. In addition, we trained a model that uses a ResNet50 feature extractor $\mathcal{F}$ pretrained on ImageNet1k [5] and uses the same MLP architecture $\mathcal{R}$ as HIPI.

S1.3 Implementation Details

Global alignment of H&E and CyCIF: We resize the CyCIF image by 2 to grossly match the H&E image resolution and use the first *Hoechst* channel for grayscaling. We find global affine registration on $\times 0.01$ downsized images using Elastix [6].

Local alignment of H&E and CyCIF: We go over the images with tiles of size 2^{14} pixels and a 2^{10} stride while allowing of 2^9 extra pixels slack in the CyCif image. We only aligned tiles that had at least 500 cells in them. To align tiles, we first downsize the tile by $\times 2^{-5}$, the find an affine registration followed by a non-linear BSpline registration implemented with SimpleITK [6]. Since the BSpline transformation is not necessarily invertable, in order to translate the cell coordinate positions in the aligned images, we iteratively search for the closest inverse coordinate for each cell. Finally, since each cell may appear in multiple tiles, we take the final cell coordinates as the mean over all tiles the cell appears in.

Extracting tile level expression: We extract image tiles of size $w = 256$ pixels with a stride of 128 together with the corresponding cells in each tile. An image tile of size 256 pixels corresponds to roughly a $128\mu m$ tissue tile. We keep only tiles that contain at least 1 cells.

Pre-trained feature extractor: We use a pre-trained ViT that uses an internal tile size of 8 pixels and has 21.7M parameters [3]. We resize image tiles to 224 pixels to match the model's design. The model outputs a feature vector of size $d = 384$. For bench-marking our model, we also used a ResNet50 feature extractor [5] pretrained on ImageNet1k with weights from TorchVision [7]. This model outputs a feature vector of size $d = 2048$ and has 25.5M parameters.

Expression regressor: We use a multi-layer perceptron with $128, 64, 32, 16$ hidden layers, a batch norm layer, a GELU activation and a 0.2 dropout layer. The model has 60.9K trainable parameters.

Sample	Keratin	Ki67	CD3	CD20	CD45RO	CD4	CD8a	CD68	CD163	FOXP3	PD1	PDL1	CD31	aSMA	Desmin	CD45
CRC01-1	60	50	51	59	50	46	53	31	48	46	43	52	40	59	55	58
CRC01-6	47	43	49	55	41	37	43	29	52	56	32	48	31	53	56	52
CRC01-13	51	46	53	57	48	50	49	36	52	45	32	43	33	59	39	54
CRC01-19	56	36	53	56	50	50	49	39	48	49	32	46	36	62	30	54
CRC01-24	59	50	56	66	52	50	45	37	50	52	29	43	37	58	34	60
CRC01-28	64	43	52	62	53	51	38	39	56	52	35	36	36	60	69	59
CRC01-33	76	58	58	62	59	52	42	40	55	64	42	57	41	65	73	64
CRC01-38	66	56	57	63	55	51	49	39	50	54	40	52	38	67	56	63
CRC01-43	73	50	61	67	61	60	48	45	63	60	46	42	49	74	53	65
CRC01-48	71	55	61	66	58	57	47	43	49	46	50	53	42	66	58	66
CRC01-53	61	56	51	57	50	45	46	35	51	50	27	49	36	54	45	59
CRC01-58	76	65	61	65	57	58	50	39	50	57	44	58	40	65	30	68
CRC01-63	76	60	63	65	58	52	56	43	51	69	11	59	42	64	61	18
CRC01-68	77	59	57	65	59	59	53	38	53	50	40	53	43	67	72	68
CRC01-73	61	56	51	55	41	44	36	40	55	43	35	55	34	54	73	51
CRC01-77	66	62	57	62	49	50	37	42	49	41	42	51	42	58	68	59
CRC01-83	68	51	57	57	51	50	38	39	47	47	46	56	41	60	77	61
CRC01-85	65	39	58	57	50	49	51	42	48	56	43	59	35	58	73	61
CRC01-90	79	66	60	68	60	55	57	40	57	59	48	59	41	69	52	67
CRC01-96	72	59	59	64	55	55	35	41	53	56	38	50	41	68	78	65
CRC01-101	70	64	58	62	55	53	47	34	48	47	50	54	36	62	77	63
CRC01-105	72	58	60	62	55	53	47	45	49	45	45	48	42	67	79	65
CRC02	56	46	12	16	41	37	33	18	26	30	39	20	26	62	45	38
CRC03	58	59	31	29	41	42	39	20	45	45	38	23	24	40	70	46
CRC12	67	70	38	41	59	44	52	22	34	27	44	19	35	38	58	57
CRC13	40	40	26	31	39	36	25	26	37	46	21	20	27	34	40	39
CRC14	57	58	46	11	46	40	25	32	40	31	29	40	15	17	60	43
CRC15	76	77	21	42	52	53	36	23	39	37	37	50	28	51	50	52
CRC17	39	50	26	53	49	42	19	17	28	16	34	25	30	52	56	53

Table S1: Top-20% accuracy. Accuracy % of HIPI in predicting the 20% of tiles with the highest expression for each sample and protein.

Training: We implemented all models and training pipelines in Pytorch. We use a base learning rate of 10^{-6}, batch size of 512 and an Adam optimizer with default parameters. We randomly augment the image tiles during training to allow for better generalization using a recent augmentation scheme *RandStainNA* designed specifically for pathology images [8]. RandStainNA is designed to constrains variable stain styles in a practicable range make the model agnostic staining intensity and normalization Furthermore, we randomly flip and rotate image tiles in contrast to the common practice on natural images. We trained models for 35 epochs and took the model checkpoint that gave the lowest loss on the validation set.

S2 Supplementary Analysis

References

[1] Mathilde Caron, Hugo Touvron, Ishan Misra, Hervé Jégou, Julien Mairal, Piotr Bojanowski, and Armand Joulin. Emerging properties in self-supervised vision transformers. In Proceedings of the IEEE/CVF international conference on computer vision, pages 9650–9660, 2021.

[2] Richard J. Chen, Chengkuan Chen, Yicong Li, Tiffany Y. Chen, Andrew D. Trister, Rahul G. Krishnan, and Faisal Mahmood. Scaling vision transformers to gigapixel images via hierarchical self-supervised learning. In Proceedings of the IEEE/CVF Conference on Computer Vision and Pattern Recognition (CVPR), pages 16144–16155, June 2022.

[3] Mingu Kang, Heon Song, Seonwook Park, Donggeun Yoo, and Sérgio Pereira. Benchmarking self-supervised learning on diverse pathology datasets, 2023.

[4] Alexey Dosovitskiy, Lucas Beyer, Alexander Kolesnikov, Dirk Weissenborn, Xiaohua Zhai, Thomas Unterthiner, Mostafa Dehghani, Matthias Minderer, Georg Heigold, Sylvain Gelly, Jakob Uszkoreit,

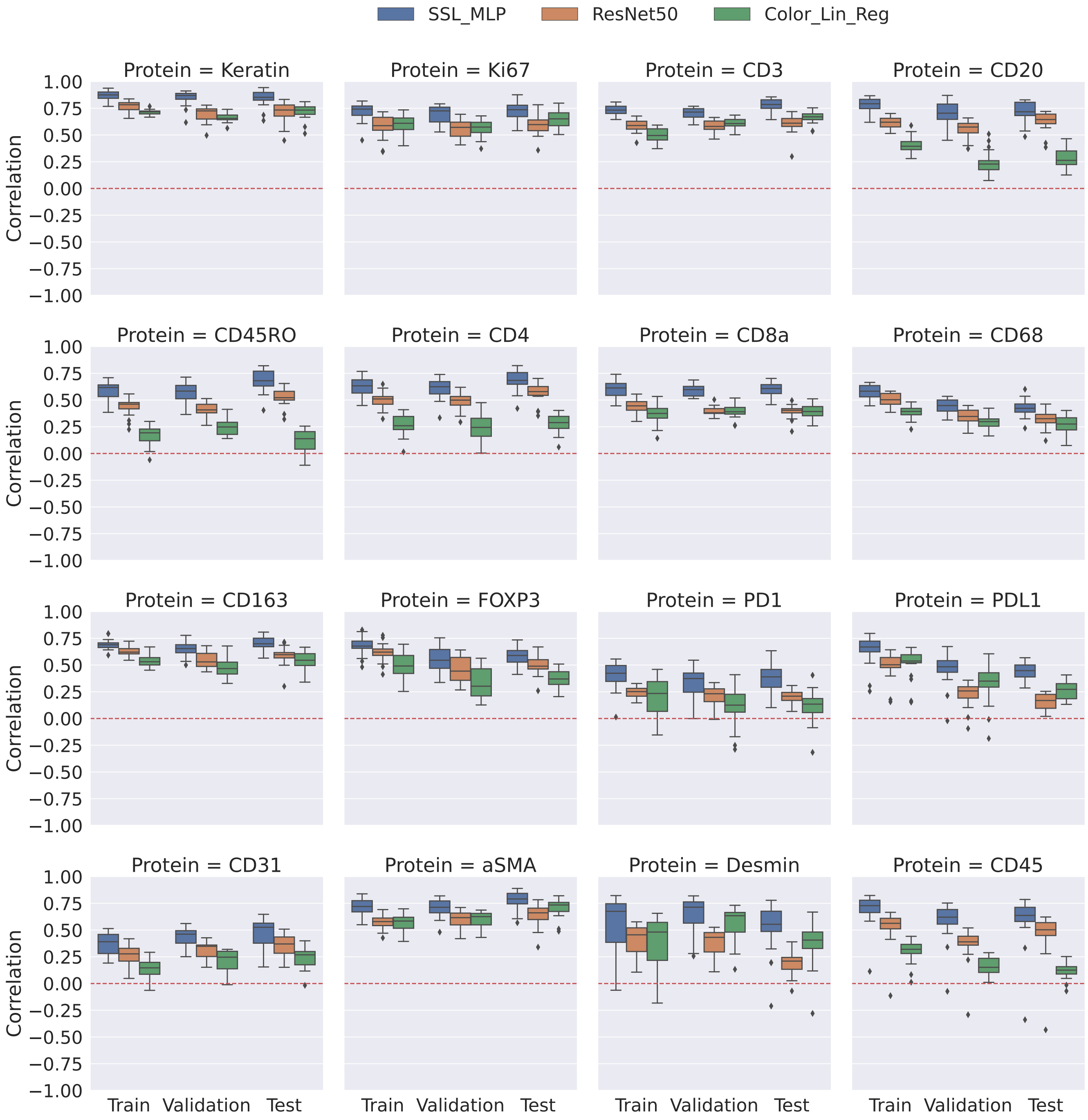

Figure S1: Correlation between measured and predicted tile level expression for 22 samples from patient CRC01 [9]. Results are divided between tiles used for training and validating models, and tiles left out for testing. Correlations for HIPI (SSL_MLP) are shown in blue, correlations for the baseline model that uses a ResNet50 feature extractor (ResNet50) are shown in orange and correlation form the baseline model that uses only the average tile colors are shown in green (Color_Lin_Reg).

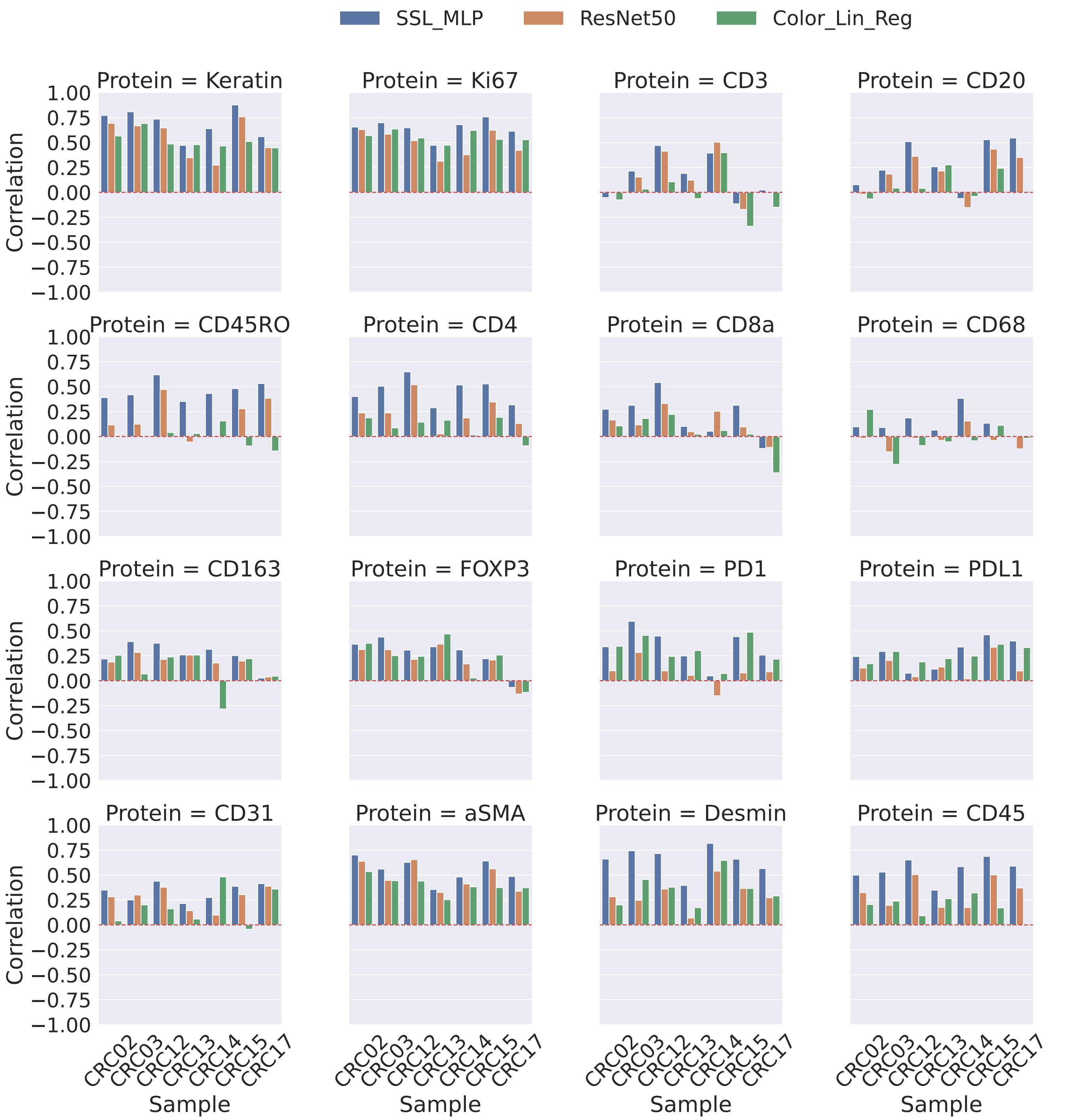

Figure S2: Correlation between measured and predicted tile level expression for patients CRC 2, 3, 12, 13, 14, 15 and 17 with different models. [9].

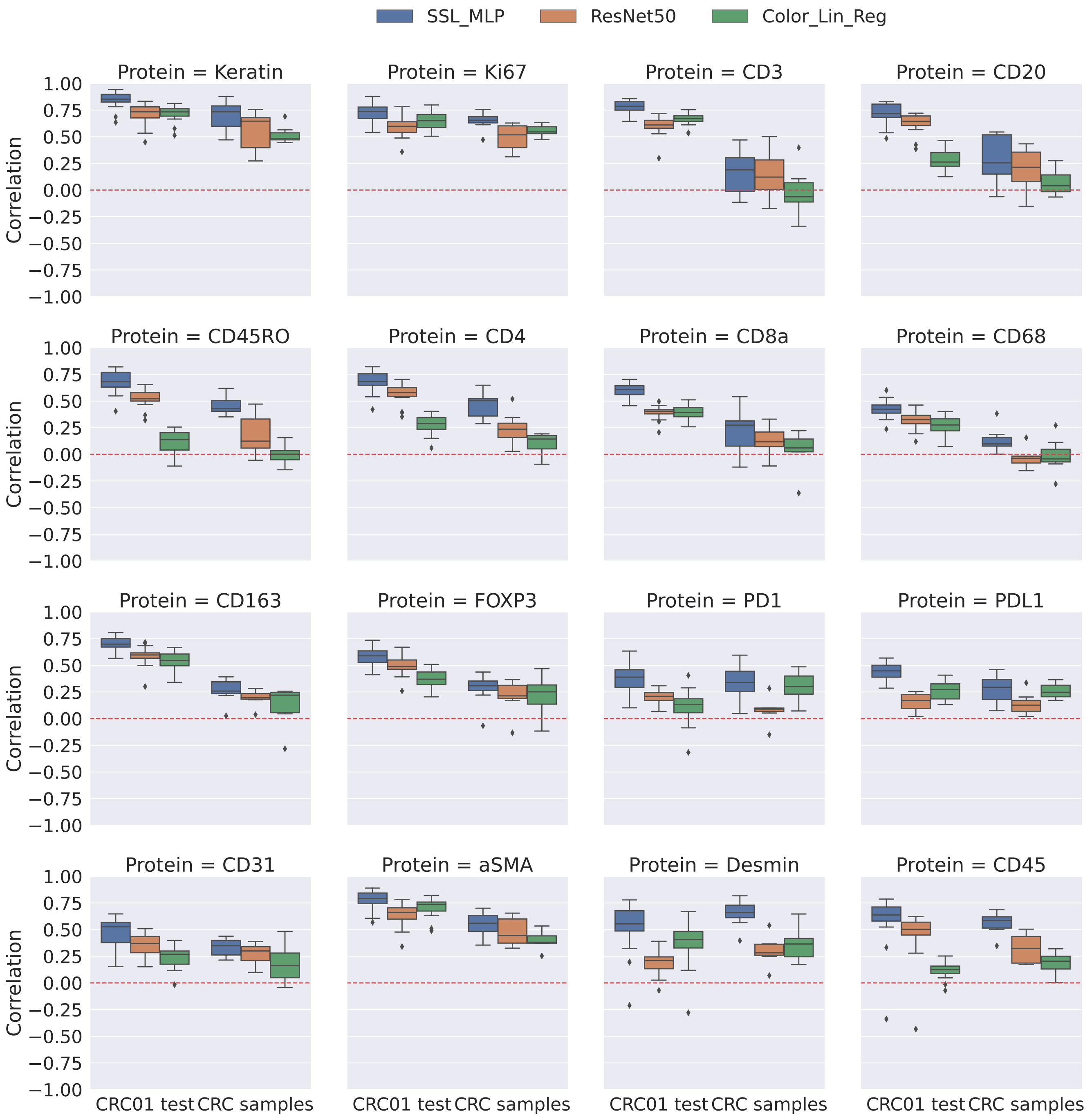

Figure S3: Correlation between measured and predicted tile level expression for test tiles of CRC01 and patients CRC 2, 3, 12, 13, 14, 15 and 17 with different models. [9].

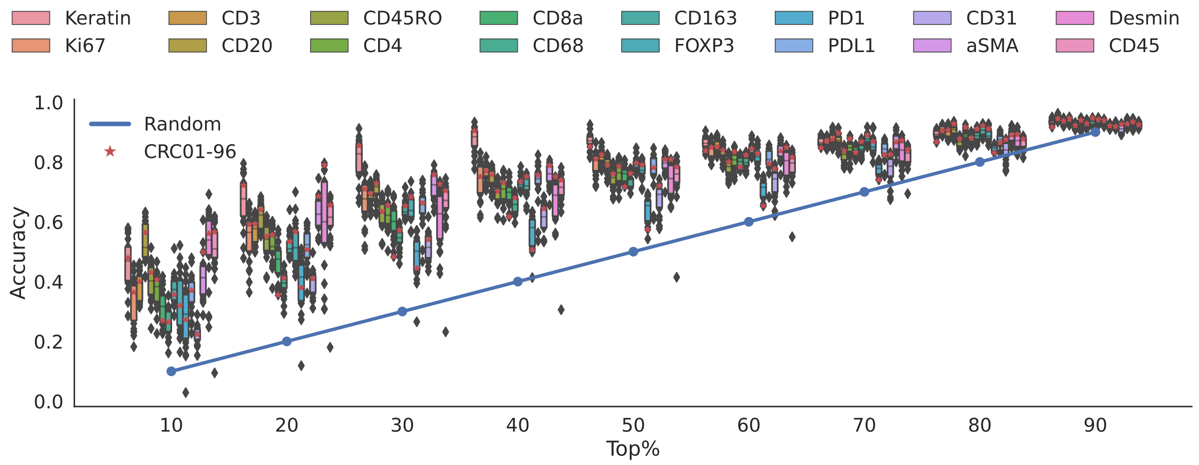

(a) CRC1 all sections

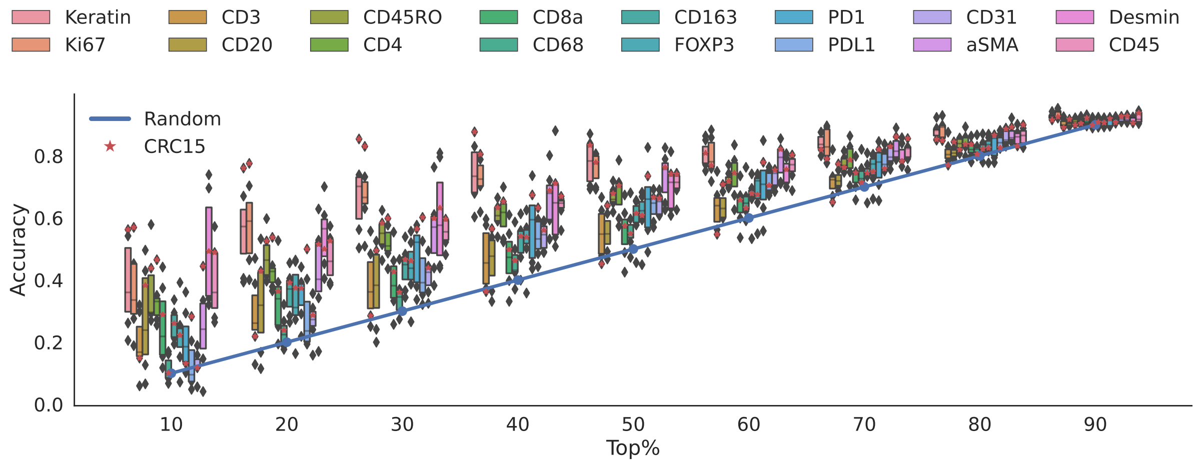

(b) CRC2-17

Figure S4: HIPI top X% accuracy. For each slide and marker, we calculate the overlap between the top X% of measured tiles and the top X% of predicted tiles and normalize by X%. (a) Slides form patient CRC1 where slide 96 is marked in red. (b) Slides from patients CRC2-17 where the slide from CRC15 is marked in red. Blue line represents the random chance accuracy.

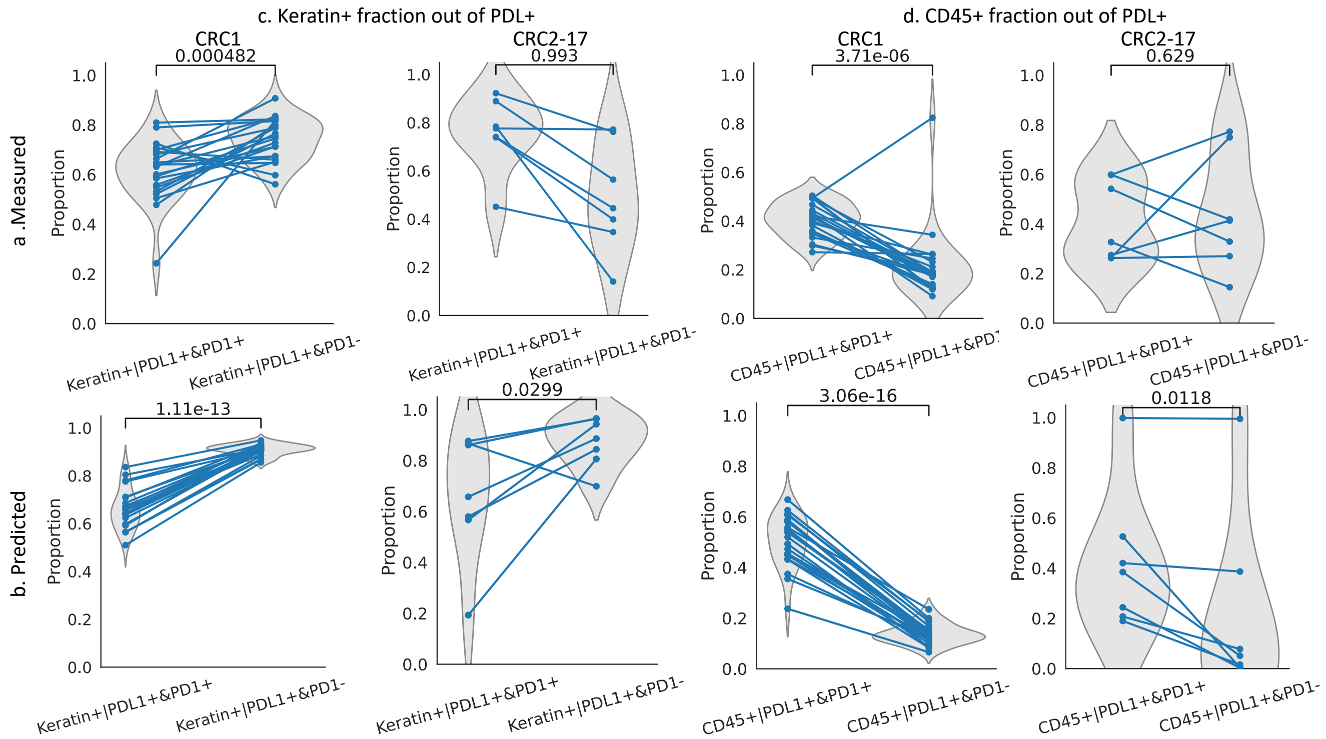

Figure S5: The proportion of Keratin+ and CD45+ tiles out of PDL1+:PD1+ interacting and non-interacting tiles. (a) Proportions observed in the processed measured tiles. (b) Proportions observed the inferred tiles. Results are summarized for each slice and presented for the slices of sample CRC1 and and slices coming from the other patients CRC2-17. The p-value of the one-sided paired t-test for the increase/decrease between PDL1+:PD1+ interacting and non-interacting tiles is presented above.

and Neil Houlsby. An image is worth 16x16 words: Transformers for image recognition at scale. In 9th International Conference on Learning Representations, ICLR 2021, Virtual Event, Austria, May 3-7, 2021. OpenReview.net, 2021.

[5] Kaiming He, Xiangyu Zhang, Shaoqing Ren, and Jian Sun. Deep residual learning for image recognition, 2015.

[6] Richard Beare, Bradley Lowekamp, and Ziv Yaniv. Image segmentation, registration and characterization in r with simpleitk. Journal of Statistical Software, 86(8):1–35, 2018.

[7] TorchVision maintainers and contributors. Torchvision: Pytorch's computer vision library. https://github.com/pytorch/vision, 2016.

[8] Yiqing Shen, Yulin Luo, Dinggang Shen, and Jing Ke. RandStainNA: Learning stain-agnostic features from histology slides by bridging stain augmentation and normalization. In Lecture Notes in Computer Science, pages 212–221. Springer Nature Switzerland, 2022.

[9] Jia-Ren Lin, Shu Wang, Shannon Coy, Yu-An Chen, Clarence Yapp, Madison Tyler, Maulik K Nariya, Cody N Heiser, Ken S Lau, Sandro Santagata, et al. Multiplexed 3d atlas of state transitions and immune interaction in colorectal cancer. Cell, 186(2):363–381, 2023.
