# Supplementary figures and images for: HIPI: Spatially resolved multiplexed protein expression inferred from H&E WSIs
Source: PLoS Comput Biol. 2024 Sep 30;20(9):e1012501. doi: 10.1371/journal.pcbi.1012501 (PMC11476684; doi:10.1371/journal.pcbi.1012501)

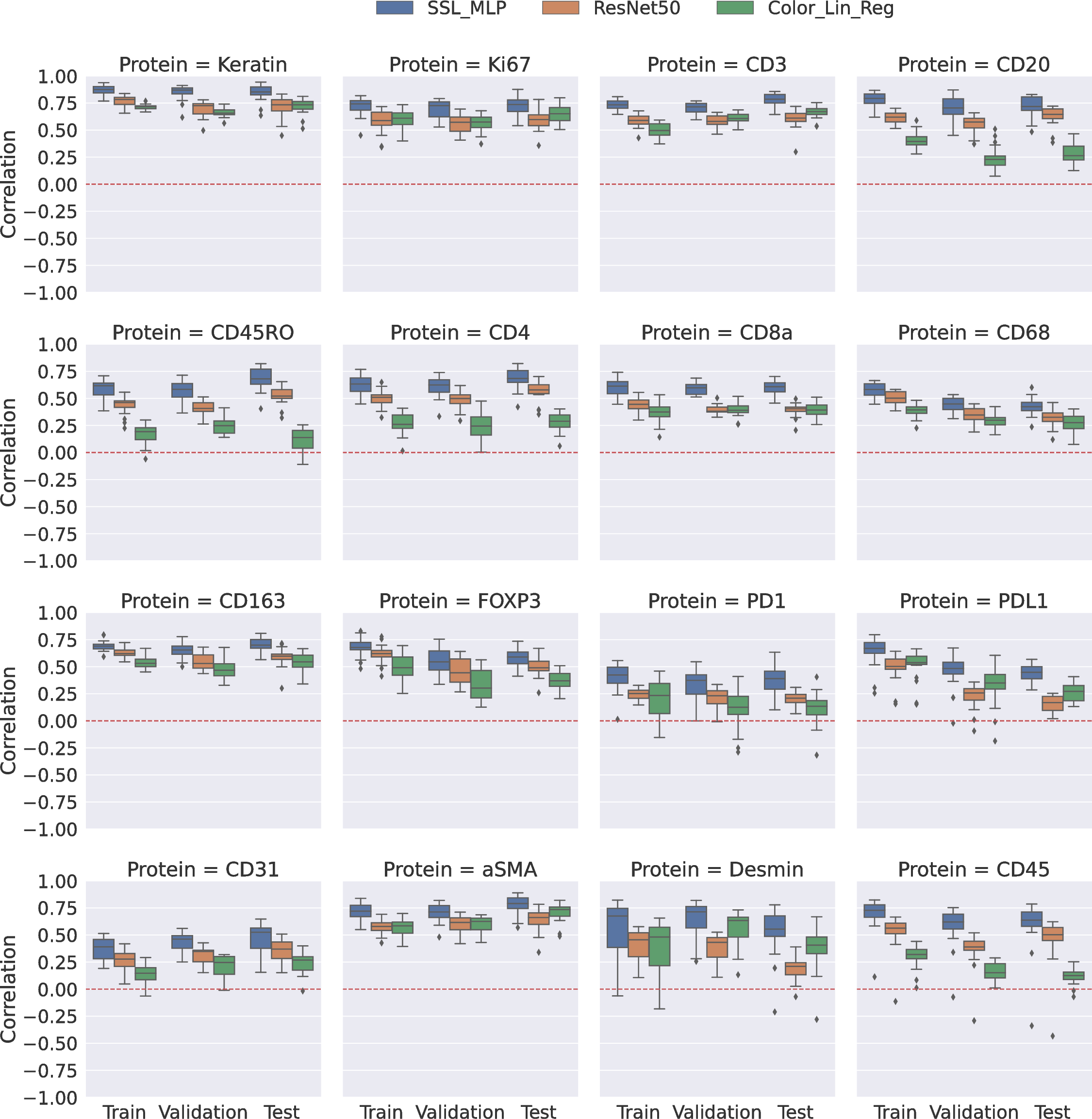

Supplement: S1 Fig — (TIF) [file pcbi.1012501.s002.tif]

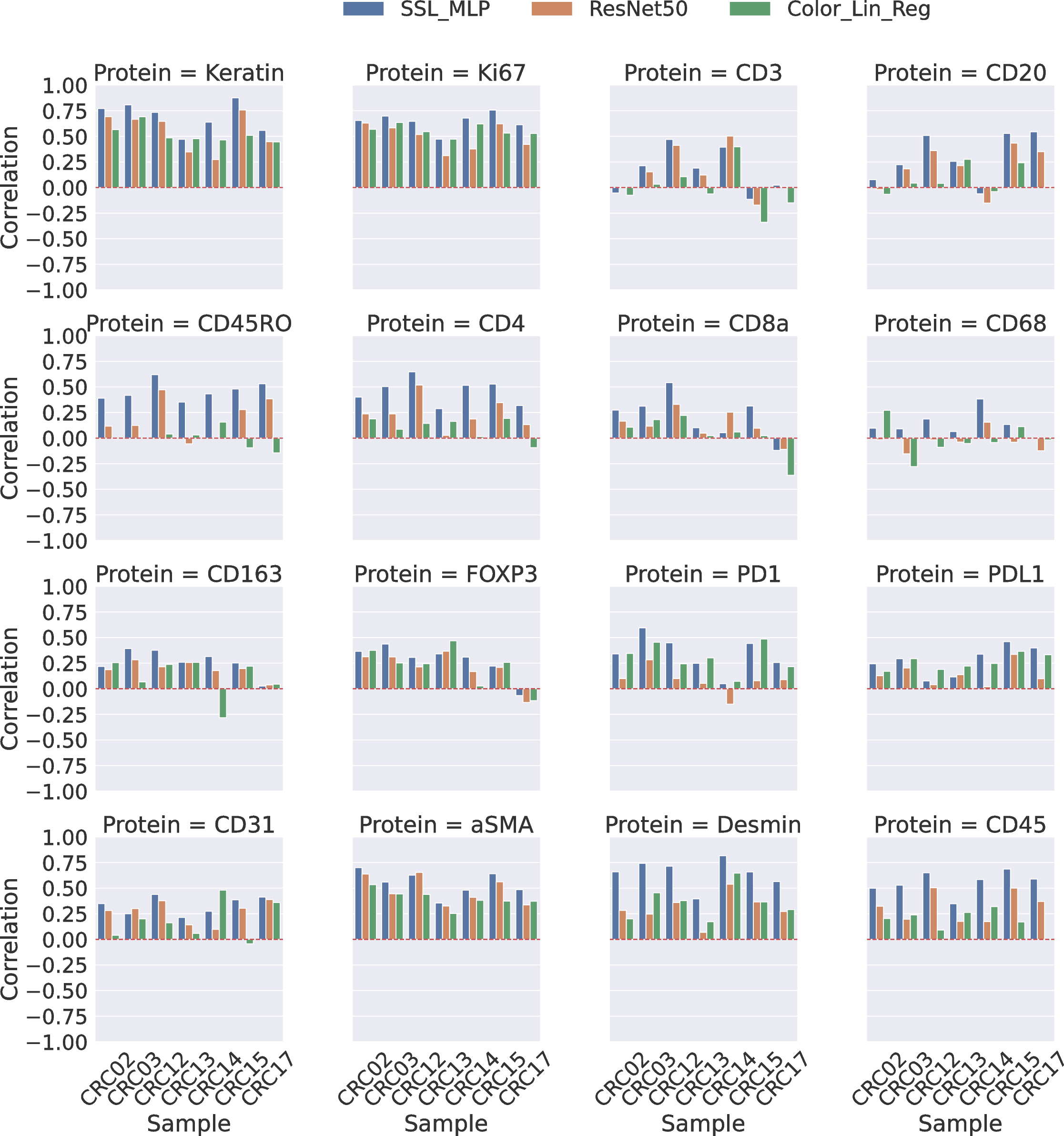

Supplement: S2 Fig — (TIF) [file pcbi.1012501.s003.tif]

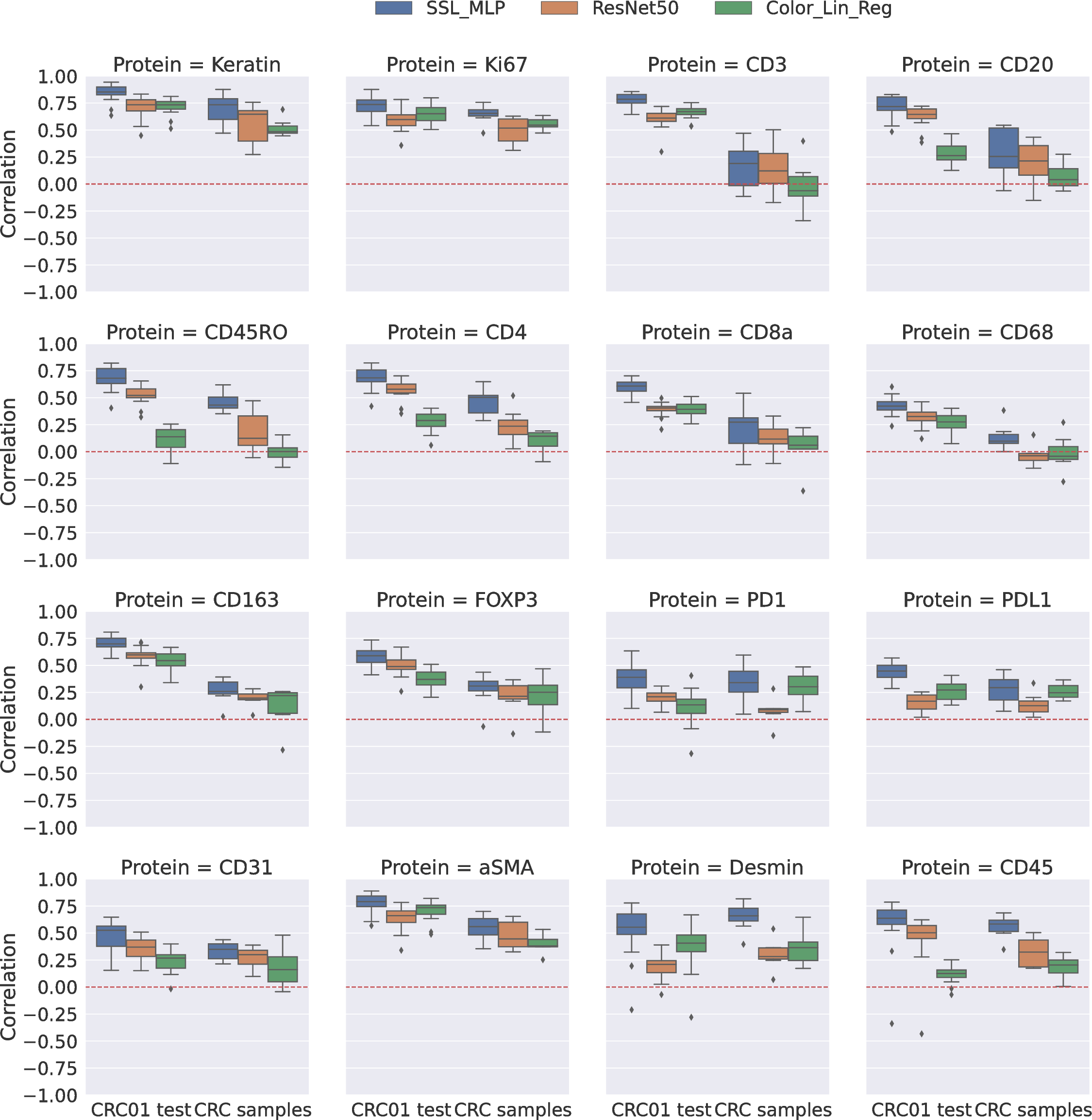

Supplement: S3 Fig — (TIF) [file pcbi.1012501.s004.tif]

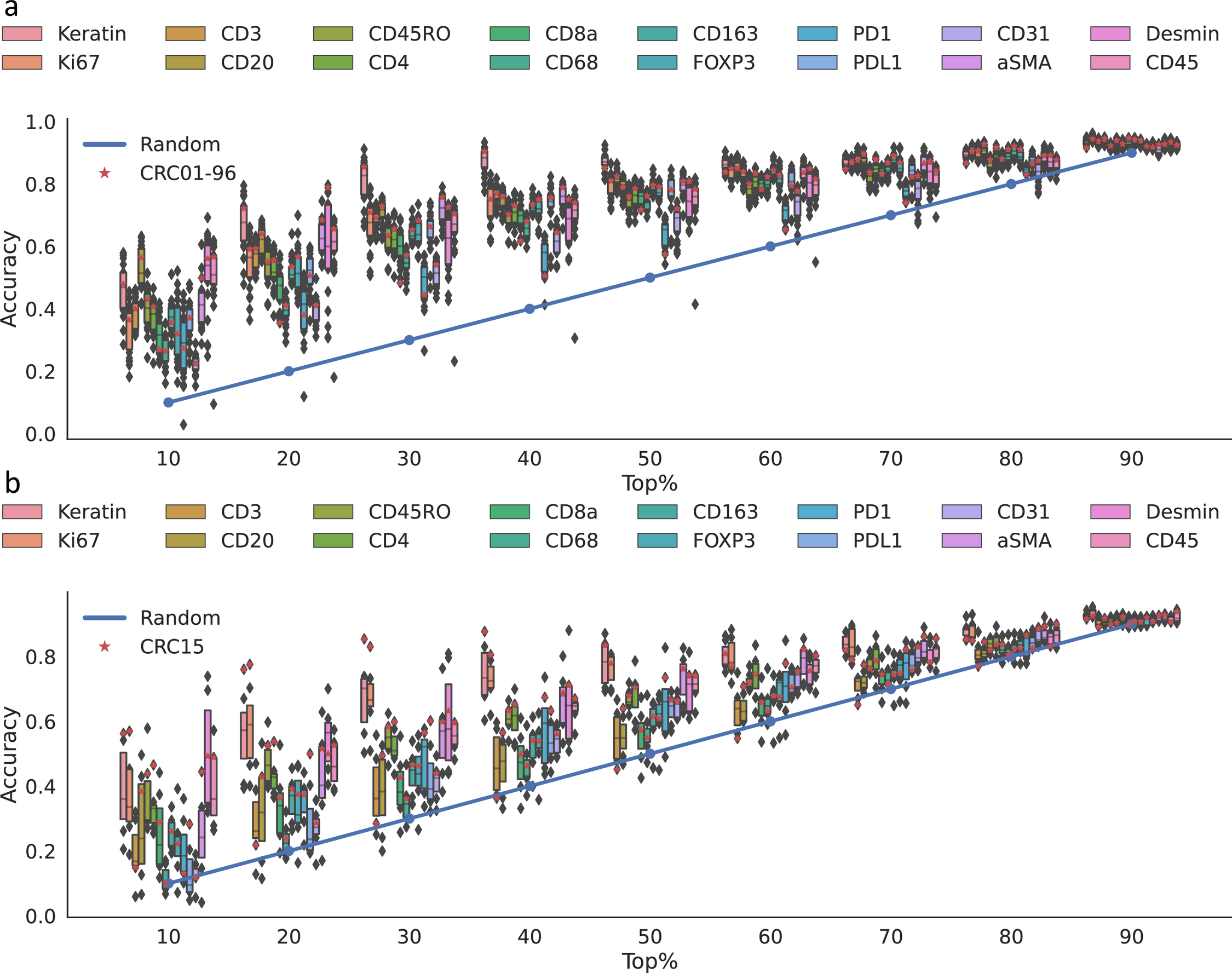

Supplement: S4 Fig — (TIF) [file pcbi.1012501.s005.tif]

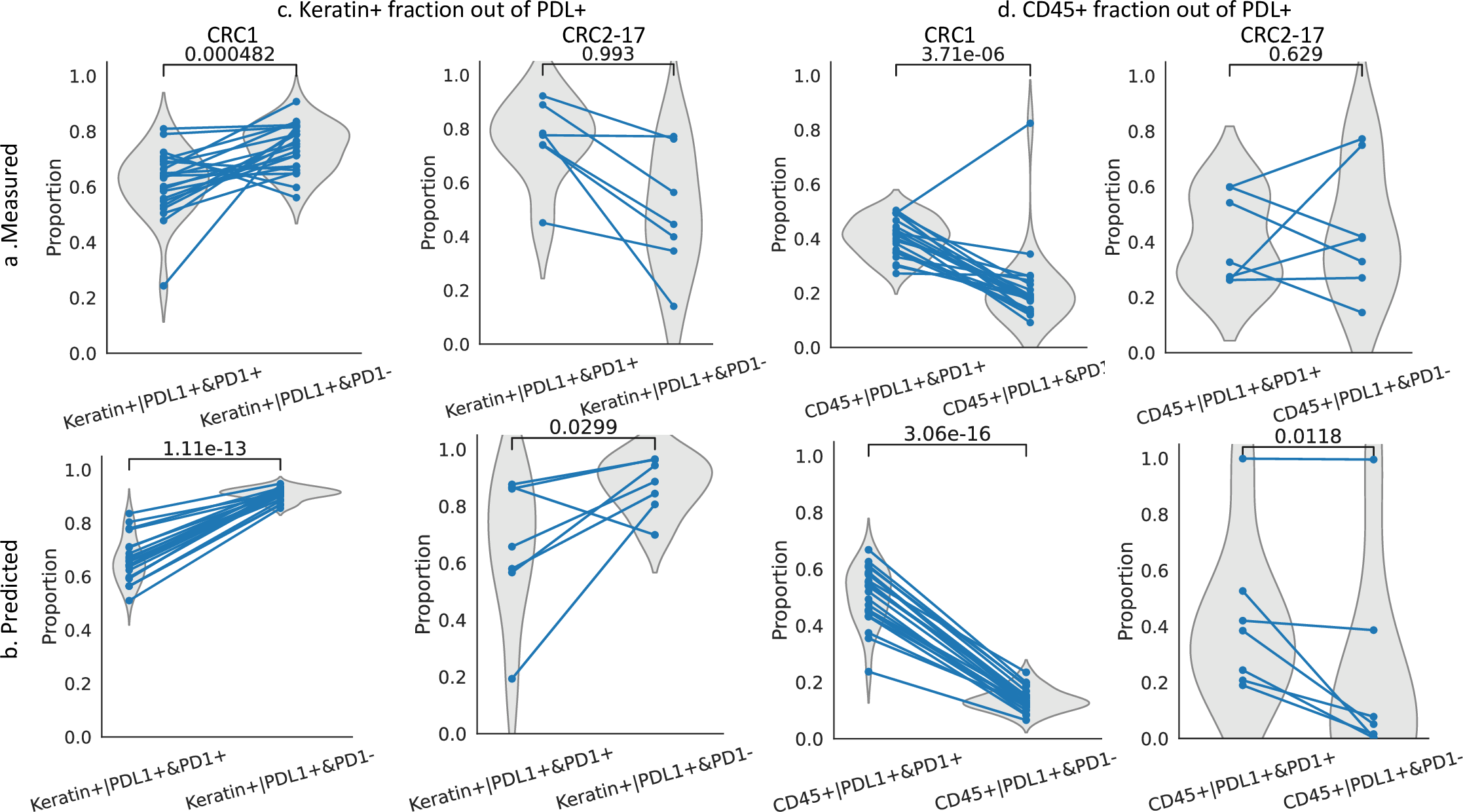

Supplement: S5 Fig — (TIF) [file pcbi.1012501.s006.tif]
