# Supplementary material for: HIPI: Spatially resolved multiplexed protein expression inferred from H&E WSIs
Source: PLoS Comput Biol. 2024 Sep 30;20(9):e1012501. doi: 10.1371/journal.pcbi.1012501 (PMC11476684; doi:10.1371/journal.pcbi.1012501)
Supplement: S1 Table — (PDF) [file pcbi.1012501.s007.pdf]

Sample	Keratin	Ki 67	CD 3	CD 20	CD45 RO	CD 4	CD 8a	CD 68	CD1 63	FOX P3	PD 1	PD L1	CD 31	aSM A	Des min	CD 45
CRC01-1	60	50	51	59	50	46	53	31	48	46	43	52	40	59	55	58
CRC01-6	47	43	49	55	41	37	43	29	52	56	32	48	31	53	56	52
CRC01-13	51	46	53	57	48	50	49	36	52	45	32	43	33	59	39	54
CRC01-19	56	36	53	56	50	50	49	39	48	49	32	46	36	62	30	54
CRC01-24	59	50	56	66	52	50	45	37	50	52	29	43	37	58	34	60
CRC01-28	64	43	52	62	53	51	38	39	56	52	35	36	36	60	69	59
CRC01-33	76	58	58	62	59	52	42	40	55	64	42	57	41	65	73	64
CRC01-38	66	56	57	63	55	51	49	39	50	54	40	52	38	67	56	63
CRC01-43	73	50	61	67	61	60	48	45	63	60	46	42	49	74	53	65
CRC01-48	71	55	61	66	58	57	47	43	49	46	50	53	42	66	58	66
CRC01-53	61	56	51	57	50	45	46	35	51	50	27	49	36	54	45	59
CRC01-58	76	65	61	65	57	58	50	39	50	57	44	58	40	65	30	68
CRC01-63	76	60	63	65	58	52	56	43	51	69	11	59	42	64	61	18
CRC01-68	77	59	57	65	59	59	53	38	53	50	40	53	43	67	72	68
CRC01-73	61	56	51	55	41	44	36	40	55	43	35	55	34	54	73	51
CRC01-77	66	62	57	62	49	50	37	42	49	41	42	51	42	58	68	59
CRC01-83	68	51	57	57	51	50	38	39	47	47	46	56	41	60	77	61
CRC01-85	65	39	58	57	50	49	51	42	48	56	43	59	35	58	73	61
CRC01-90	79	66	60	68	60	55	57	40	57	59	48	59	41	69	52	67
CRC01-96	72	59	59	64	55	55	35	41	53	56	38	50	41	68	78	65
CRC01-101	70	64	58	62	55	53	47	34	48	47	50	54	36	62	77	63
CRC01-105	72	58	60	62	55	53	47	45	49	45	45	48	42	67	79	65

Sample	Keratin	Ki67	CD3	CD20	CD45RO	CD4	CD8a	CD68	CD163	FOXP3	PD1	PDL1	CD31	aSMA	Desmin	CD45
CRC02	56	46	12	16	41	37	33	18	26	30	39	20	26	62	45	38
CRC03	58	59	31	29	41	42	39	20	45	45	38	23	24	40	70	46
CRC12	67	70	38	41	59	44	52	22	34	27	44	19	35	38	58	57
CRC13	40	40	26	31	39	36	25	26	37	46	21	20	27	34	40	39
CRC14	57	58	46	11	46	40	25	32	40	31	29	40	15	17	60	43
CRC15	76	77	21	42	52	53	36	23	39	37	37	50	28	51	50	52
CRC17	39	50	26	53	49	42	19	17	28	16	34	25	30	52	56	53
