# Supplementary material for: HIPI: Spatially resolved multiplexed protein expression inferred from H&E WSIs
Source: PLoS Comput Biol. 2024 Sep 30;20(9):e1012501. doi: 10.1371/journal.pcbi.1012501 (PMC11476684; doi:10.1371/journal.pcbi.1012501)
Supplement: Attachment — Submitted filename: response to reviewers RECOMB-CCB 2024 paper 604.pdf [file pcbi.1012501.s008.pdf]

SUBMISSION: 604
TITLE: HIPI: Spatially Resolved Multiplexed Protein ExpressionInferred from H&E WSIs

---------------------- REVIEW 1 ---------------------
SUBMISSION: 604
TITLE: HIPI: Spatially Resolved Multiplexed Protein ExpressionInferred from H&E WSIs
AUTHORS: Ron Zeira, Leon Anavy, Zohar Yakhini, Ehud Rivlin and Daniel Freedman

----------- Overall evaluation -----------
SCORE: 2 (accept)
----- TEXT:
The authors introduce a new computational method, HIPI, for predicting CyCIF protein expression from H&E WSIs. HIPI combines a self-supervised pre-trained model for feature extraction with a fully connected multi-layer perceptron for regression on the extracted features.

The authors first trained and tested HIPI on 22 pairs of CyCIF and H&E WSIs from a single colorectal cancer patient (but with adequately partitioned training and test sets), and they then tested HIPI again on samples from 7 new colorectal cancer patients. They generally observed high positive correlations between the observed and predicted values for 16 different protein markers with some variation between markers and performance on the training and test sets. They also included a comparison with a baseline model with much simpler features (mean color intensities) and observed much lower positive correlations.

The manuscript is clear and well written, and experimental design is reasonable, and the results are convincing and clinically relevant. I noticed several mostly minor issues:
1. The repository provides links to data and code for other projects, but it does not appear to provide code for HIPI. While the manuscript provides many of the details of the method, and many readers should be able to implement a close approximation of the method, code and examples from the manuscript are always helpful for improving the usability and reproducibility of the research in the manuscript.
We have published all the code for processing the H&E and CyCIF data, training and evaluating the model, as well as the trained model weights: https://github.com/RonZeira/HIPI/
2. By design, the baseline comparison is simple, but perhaps too simple to separate the contributions from the feature extraction and regression components of the model.
We appreciate the reviewer’s helpful suggestion. We trained another model that uses a ResNet50 feature extractor pre-trained on ImageNet1k and the same prediction head architecture as our model. This comparison evaluates our use of a pathology specific self supervised pretrained feature extractor in contrast to some general vision feature extractor. We saw that our model outperformed the ResNet model on all markers and tissue slices further supporting our claim (Supplementary figures S1, S2 and S3).
3. It would be interesting to see how image quality and other artifacts affect the results, to consider transfer learning for different cancer types, etc.

We agree with the reviewer that artifacts such as image quality and staining intensity can affect the results. To address this, during training, we employ an image augmentation scheme called RandStainNA (Shen *et al*. 2022) which constrains variable stain styles in a practicable range in order to train a stain agnostic model. In addition, we perform image flips and rotations during training to prevent the model from learning the tissue orientation. We further emphasize this in the text.
We indeed think that transferring the model to other cancer types is a valuable feature. However, supporting multiple cancer types requires additional training on the appropriate organs as different tumors may express different proteins. Unfortunately, we did not find additional datasets that contained multiple paired WSI and CyCIF measurements from other cancer types. We address these issues in our discussion.
3. Again, the manuscript is extremely clear, but there are some minor typos, including "multiple task" and "tileto".
We fixed these typos as well as others we found.
4. Some of the text in most of the figures is illegibly small, especially when printed or on a small screen. Please enlarge the text to be legible, or shrink the size of the figure when plotting it so that the text is larger relative to the figure elements.
We thank the reviewers for their suggestion. We increased the fonts on all figures and changed some of the figure's layout to improve readability.

----------------------- REVIEW 2 ---------------------
SUBMISSION: 604
TITLE: HIPI: Spatially Resolved Multiplexed Protein ExpressionInferred from H&E WSIs
AUTHORS: Ron Zeira, Leon Anavy, Zohar Yakhini, Ehud Rivlin and Daniel Freedman

----------- Overall evaluation -----------
SCORE: 1 (weak accept)
----- TEXT:
In this paper, the authors proposed a deep learning-based model to predict the protein expression level from H&E images. The training and test sets are generated by aligning the H&E section and its adjacent section probed by CyCIF. Here, the model architecture is a Vision transformer (ViT), initialized with pre-trained parameters. The model itself is the mainstream and state-of-the-art method, and the task of predicting the CyCIF protein is new. The manuscript itself is overall well written.

Here are a few specific comments:

1. in Figures 2-5, the authors only presented the selected results, which may lead to overclaim or misinterpretation. Please present the results of all proteins in the main figures, possibly in an aggregated form.
We added panels to figures 2 and 3 showing the distribution of Pearson correlations between measured and predicted values across all slices and all markers on the test set. More elaborate

presentation of the full results are given in supplementary figures S1, S2 and S3. We chose to present selected markers that have biological/clinical significance in the main figures. In the figures, we have also included markers for which the model exhibited lower performance, specifically PD1 and PDL1, as we mention in the text.

2. the baseline models are too simple. There are multiple methods to use H&E image to predict gene expression (STNet, DOI: 10.1038/s41551-020-0578-x; BLEEP, DOI: 10.48550/arXiv.2306.01859) or cell type (Hist2Cell, DOI: 10.1101/2024.02.17.580852). All of them used evaluation by hold-out dataset, e.g., leave-one-slide/donor-out. P.S., in the Discussion (the first point), the author motioned XFuse, but it aims for imputation (i.e., higher resolution) not for prediction, so it requires an in-sample setting.

We agree with the reviewer that the baseline model that uses only the average tile colors is very simple, although performing relatively well. Nevertheless, per the reviewers suggestions we also trained another model that uses a ResNet50 feature extractor pre-trained on ImageNet1k and the same prediction head architecture as our model. This comparison evaluates our use of a pathology specific self supervised pretrained feature extractor in contrast to some general vision feature extractor. We saw that our model outperformed the ResNet model on all markers and tissue slices further supporting our claim (Supplementary figures S1, S2 and S3).

We thank the reviewer for pointing out other methods that aim to predict molecular features from H&E images. However, we did not find a way to easily compare our model to these methods. Some methods are designed for spatial transcriptomics technology which measure sparse RNA expression with a few thousands ~100μm spots and therefore a direct comparison is not possible. Other methods try to predict cell type, again making it hard to compare to.

3. the use of adjacent sections to generate ground truth is reasonable, while it looks like there is new technology supporting the same section (DOI: 10.1038/s43018-023-00576-1), please consider if this may enhance the quality of the ground truth.

We thank the reviewer for turning our attention to this paper. Using such technology may indeed enhance the quality of the ground truth as it removes the need for aligning adjacent tissue slices. We hope to utilize this dataset in the near future. Notice however that even without perfect alignment between adjacent slices, our method is still able to give good results.

4. please consider moving the within-slide-split results to supplementary figures, as it may over-present the performance. In real practice, it generally applies to a new slide.

We changed the structure of figures 2 and 3 and added panels showing the distribution of Pearson correlations between measured and predicted values across all slices and all markers on the test sets.

5. it can be helpful to add more details about the model architecture and training procedure.

We added more details describing the model in section 2.1. We left the full explicit details on the model, training and implementation in the supplementary methods section S1. In addition, we published the code on GitHub.

----------------------- REVIEW 3 ---------------------

SUBMISSION: 604

TITLE: HIPI: Spatially Resolved Multiplexed Protein ExpressionInferred from H&E WSIs

AUTHORS: Ron Zeira, Leon Anavy, Zohar Yakhini, Ehud Rivlin and Daniel Freedman

----------- Overall evaluation -----------
SCORE: -2 (reject)
----- TEXT:
The authors introduce their approach "HIPI: Spatially Resolved Multiplexed Protein Expression Inferred from H&E WSIs". First of all, it is unfortunate to use abbreviations in the title. Both H&E as well as WSI will be non-common abbreviations to most readers.

They present a deep-learning model that predicts protein expression levels/cellular markers from H&E staining images. This is a potentially useful computational procedure as H&E images are more standard than the more expensive protein expression measurements. They show good results on a small colorectal cancer data set. I am missing, however, a comparison to alternative approaches, the authors mention [15,16,17,19] as related work. So it is hard to judge the added value of the new approach over existing methods.
We trained another model for comparison that uses a ResNet50 feature extractor pre-trained on ImageNet1k and the same prediction head architecture as our model. This comparison evaluates our use of a pathology specific self supervised pretrained feature extractor in contrast to some general vision feature extractor. We saw that our model outperformed the ResNet model on all markers and tissue slices further supporting our claim (Supplementary figures S1, S2 and S3).
We did not find a way to easily compare our model to these methods. These methods are designed for spatial transcriptomics technology which measure sparse RNA expression with a few thousands ~100µm spots and therefore a direct comparison is not possible.

In particular, I am also missing a better description of the method which is currently only sketched in Fig. 1, which is briefly accompanied by a short text in the supplement. In my opinion, a RECOMB submission should have a strong focus on the method and its novelty.
We added more details describing the model in section 2.1. We left the full explicit details on the model, training and implementation in the supplementary methods section S1.

My main criticism is, however, that the code is not available. This makes the work irreproducible and therefore, in my opinion, not aceptable for RECOMB-CCB.
We have published all the code for processing the H&E and CyCIF data, training and evaluating the model, as well as the trained model weights: https://github.com/RonZeira/HIPI/
