# Supplementary material for: HIPI: Spatially resolved multiplexed protein expression inferred from H&E WSIs
Source: PLoS Comput Biol. 2024 Sep 30;20(9):e1012501. doi: 10.1371/journal.pcbi.1012501 (PMC11476684; doi:10.1371/journal.pcbi.1012501)
Supplement: Attachment — Submitted filename: PCOMPBIOL-D-24-01094 response to reviewers.pdf [file pcbi.1012501.s009.pdf]

PCOMPBIOL-D-24-01094 - Response to reviewer comments

The original text of the reviewer comments are kept in black and we mark our responses in red.

Reviewer #1: The authors have satisfactorily addressed the comments from my previous review, with two minor exceptions.

1. The authors have provided their code, which is important and appreciated. I would encourage them to update the README in their GitHub repository to provide example commands for their entire algorithmic pipeline. Example commands for the training and inference steps are provided, but not for the data processing steps.

We thank the reviewer for pointing this out. We have updated our preprocessing scripts and the repo's README to provide further explanations on the preprocessing steps.

2. The authors have updated their figures to make them more legible, but there are still parts with small text that are difficult to read, namely, the color bars in Figures 2 and 3 and the legends in Figures 4 and 5. Unfortunately, the figures are not vector graphics, but, fortunately, they appear to be high-resolution raster graphics that can be enlarged on a computer screen and more easily read. I would encourage them to enlarge the small text in the figures so that it can be read by most people on a printed sheet.

We have updated figures 2-5 per the reviewer's suggestion with an updated layout and larger fonts.

Reviewer #2: I am the reviewer #2 from RECOMB. I thank the authors for replying to my comments and adding more analyses. Overall, I think this would be valuable work but some changes in the presentation may help avoid over-presenting the performance, therefore I would further clarify my original comments 3 & 4.

3. While using the consecutive section seemingly works well, it still has risks of over-connecting the two modalities during the registration. Therefore, if possible, please

consider using at least one slide from the technology supporting within the slide (proteins and H&E) as a test set.

We thank the reviewer for this suggestion. However, we did not find it possible to use this new technology in our current research. We note that there are some differences in technology in comparison to the CyCIF data we used to train our model which can affect results even without taking into account the registration issues. Furthermore, the suggested technology captures a different set of markers making a direct comparison limited. Therefore we think that comparing this technology in the current setting would require significant effort. In addition, in regard to the risk of over-connecting the two modalities during registration, we want to emphasize that we only use one of the autofluorescence channels for registration and not any of the protein marker channels.

4. The new supp figures S2 and S3 strongly suggest that there is a substantial decrease when applying to external datasets. I'm not saying the performance is too low in the external datasets; it is still within a reasonable range, considering the limited accuracy in predicting RNA levels in spatial transcriptomics data. However, the much higher performance in the within-slide test set can be over-confident about the prediction, as the framework was proposed to predict the unseen H&E tissues, instead of unseen regions of the tissue. Please re-arrange the results by presenting the cross-slide or cross-sample prediction as the main figure.

We have updated Figure 3c to include comparison between the held out sample and the test set from the main training sample, as suggested by the reviewer. In the main text, we discuss the performance differences between the test set left out of training from the first sample and the external sample test set.

In addition to the reviews you will see below, we have asked reviewers 1 and 2 to comment on your reply to reviewer 3, who was not available to re-review the manuscript. In your revision, please address these additional points as well:

Reviewer 1:

I believe the authors have satisfactorily addressed Reviewer 3's comments.

The first comment is that the authors do not compare against existing methods, but the authors correctly note that the methods are different, and a direct comparison to these methods is not informative. They do add a comparison with other methods, partially in response to other reviewer feedback. More comparisons with existing methods would be welcome, but are not informative if they address different tasks.

The second comment is that the authors do not describe the method adequately. It is described appropriately.

The third comment is that the authors do not share their code. This was a significant problem that the other reviewers noted, but they have now shared their code.

Reviewer 2:

I had a quick look on the response to reviewers 3 and here are the comments for the 3 points, accordingly:
1) the benchmarking methods are indeed a good question. The authors now added more baseline methods, which are good, but saying existing RNA-prediction methods are readily applicable. This is true but not undoable at all.
2) the methodology is indeed mainly within the supp S1. While concise, it looks major information is included. The authors may need to structure it to align with the journal format and for easier reading.
3) it looks the codes for the models are available but the analysis notebooks are not. It will be very helpful to ensure reproducibility if the authors can also release the analysis notebooks/scripts.
